# Tris[2,2,6,6-tetra­methyl-8-(tri­methyl­sil­yl)benzo[1,2-*d*;4,5-*d*′]bis­(1,3-di­thiol)-4-yl]methanol diethyl ether monosolvate

**DOI:** 10.1107/S2056989018004516

**Published:** 2018-03-23

**Authors:** Nico Fleck, Gregor Schnakenburg, Alexander C. Filippou, Olav Schiemann

**Affiliations:** a University of Bonn, Institute of Physical and Theoretical Chemistry, Wegelerstrasse 12, 53115 Bonn, Germany; b University of Bonn, Institute of Inorganic Chemistry, Gerhard-Domagk-Strasse 1, 53121 Bonn, Germany

**Keywords:** crystal structure, tri­aryl­methanol, trityl radical, EPR, spin label

## Abstract

The title compound, a tri­aryl­methanol, C_46_H_64_OS_12_Si_3_ **1**, was synthesized *via* li­thia­tion of tris-2,2,6,6-tetra­methyl­benzo[1,2-*d*;4,5-*d*′]bis­[1,3]di­thiol-4-yl-methanol, **2**, and electrophilic quenching with tri­methyl­silyl chloride. The current crystal structure reveals information about the reactivity of this compound and compares well with the structure reported for the unsubstituted parent compound **2** [Driesschaert *et al.* (2012 ▸). *Eur. J. Org. Chem.* **33**, 6517–6525]. The title compound **1** forms mol­ecular propellers and crystallizes in *P* , featuring an unusually long Si—C_ar_ bond of 1.910 (3) Å. Moreover, the geometry at the central quaternary carbon is rather trigonal-pyramidal than tetra­hedral due to vast intra­molecular stress. One tri­methyl­silyl group is disordered over two positions in a 0.504 (4):0.496 (4) ratio and one S atom is disordered over two positions in a 0.509 (7):0.491 (7) ratio. The contribution of disordered diethyl ether solvent mol­ecule(s) was removed using the *PLATON* SQUEEZE (Spek, 2015 ▸) solvent masking procedure. These solvent mol­ecules are not considered in the given chemical formula and other crystal data.

## Abstract 2

The title compound is a precursor of a stable tri­aryl­methyl radical used in EPR-spectroscopy. It’s structure features a propeller-like conformation of the phenyl rings and a sterically crowded geometry at the central carbon.

## Chemical context   

The reported tri­aryl­methanol **1** is the direct precursor of the corresponding tri­aryl­methyl radical. Such tetra­thi­aryl­methyl radicals, also called trityl radicals, can be used as spin labels for EPR-based distance measurements (Reginsson *et al.*, 2012 ▸; Kunjir *et al.*, 2013 ▸) and have recently been employed for structure determination in proteins (Jassoy *et al.*, 2017 ▸; Yang *et al.*, 2012 ▸) as well as nucleic acids (Shevelev *et al.*, 2015 ▸). They are also used for dynamic nuclear polarization experiments (Jähnig *et al.*, 2017 ▸). Trityl radicals feature a very narrow linewidth in EPR spectra, slow spin–spin relaxation at room temperature and show line-broadening depending on the oxygen concentration in their surroundings. The latter property also makes them suitable as oxygen probes (Frank *et al.*, 2015 ▸). However, most of the trityl radicals reported in the literature feature carb­oxy­lic acid derivatives as substituents in the *para*-position. The title compound **1** is a promising precursor for differently *para*-substituted trityl alcohols and their corresponding radicals.

## Structural commentary   

**Figure 1:**
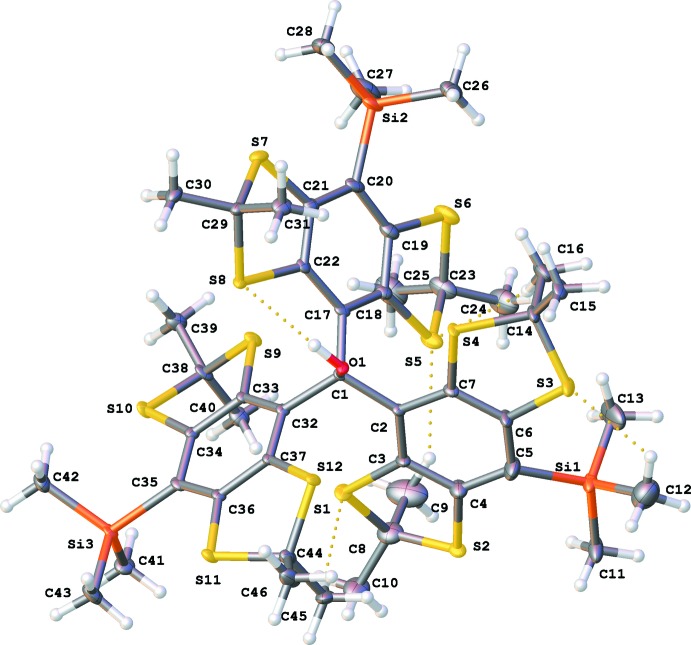
Crystal structure of the title compound, **1**. Displacement ellipsoids are at the 50% probability level. Only the major disorder component is shown.

Compound **1** crystallizes (in space group *P* 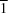 with the unit cell containing two mol­ecules) from diethyl ether as a racemic mixture with respect to the propeller-like conformation of the aryl building blocks. The unit cell consists of one *P*- and one *M*-configured mol­ecule, as shown in Fig. 1 ▸.
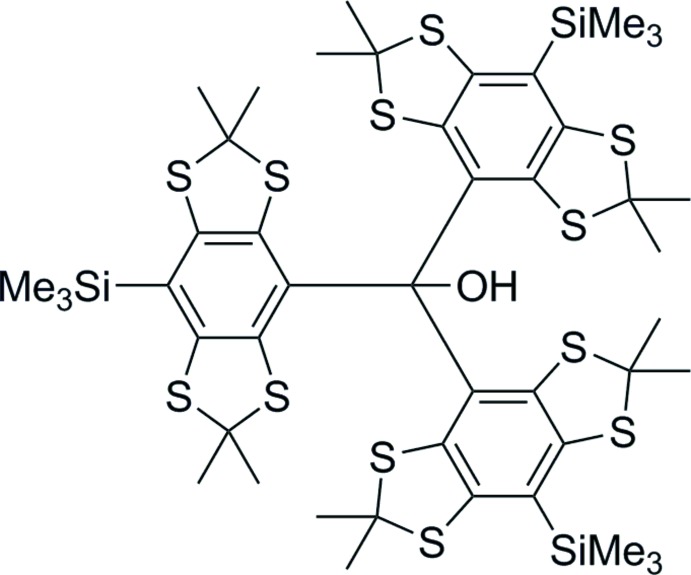


**Figure 2:**
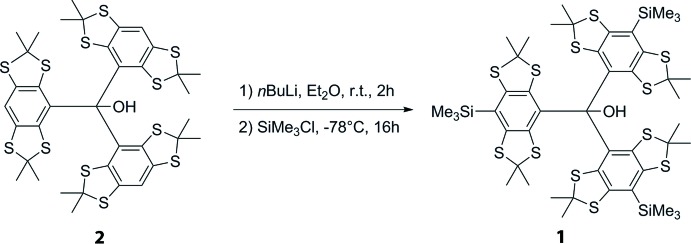
Synthesis of the title compound **1**.

The structure of the title compound deviates from *C*_3_ symmetry, since the dihedral angles between the aryl planes are not equivalent (±73.7, ±73.7, ±70.2°). Moreover, the structure of **1** exhibits an Si—C_ar_ bond length of 1.909 (3) Å to 1.945 (4) Å, whereas a bond length *X*_3_Si—C_ar_ of 1.863 (14) Å is typically expected (Allen *et al.*, 1987 ▸). This elongation of the Si—C_ar_ bond may be due to the sterical stress at the *para*-positions caused by vicinal sulfur atoms. Additionally, the bond angles between the tetra­thiaryl substituents at C1 are 112.2 (2), 113.5 (2) and 114.0 (2)°, exceeding the tetra­hedral angle of 109.5°. Therefore, regarding its geometry, C1 is situated between a tetra­hedral and a trigonal–planar environment with a deviation of 0.409 (4) Å from the plane through atoms C2, C17 and C32. This coincides with the experimental observation that the title compound forms the corresponding carbocation with low effort, meaning its structure is already similar to the transition state according to Hammond’s postulate. However, the C1—O1 bond length of 1.439 (3) Å fits the value expected for tertiary alcohols, which is 1.440 (12) Å (Allen *et al.*, 1987 ▸) and does not show any elongation. Regarding the envelope-configured 1,3-di­thia­nes, C—S—C angles between 94.4 (2) and 96.1 (2)° and C—C—S—C torsion angles in 1,3-di­thia­nes between 18.7 and 26.9° are observed, with the methyl­ene groups pointing either above or below the aromatic ring plane although without regularity. This is also observed within the crystal structure of the unsubstituted trityl alcohol **2** (Fig. 2 ▸).

The mol­ecular structure of compound **1** features an O1—H1⋯S8 hydrogen bond with a donor to acceptor atom distance of 3.031 (2) Å, which falls into the regime of a moderately strong hydrogen bond according to Jeffrey (1997 ▸). In addition, the H1⋯S8 distance of 2.32 Å is significantly shorter than 2.90 Å, the sum of the van der Waals radii (Bondi, 1964 ▸). The remaining five intra­molecular hydrogen bonds listed in Table 1 ▸ belong into the category of weak electrostatic hydrogen bonds, with the shortest having a donor–acceptor atom distance of 3.435 (3) Å and the longest a donor–acceptor distance of 3.926 (5) Å. Other contacts between the mol­ecules were not observed.

## Supra­molecular features   

In the crystal, a number of C—H⋯S inter­actions occur (Table 1 ▸).

## Database survey   

The Cambridge Structural Database (CSD, Version 5.38; Groom *et al.*, 2016 ▸) contained two structures of *para*-substituted trityl radicals [ESECUB (Decroos *et al.*, 2011 ▸) and TIXCEJ (Liu *et al.*, 2008 ▸)] and one structure determination for compound **2** (REGBUG; Driesschaert *et al.*, 2012 ▸). As found here for compound **1**, the reported structure of **2** also deviates from *C*_3_ symmetry, with dihedral angles for the aryl planes of ±75.3, ±70.7, ±69.9°. However, in contrast to the crystal structure reported here, Driesschaert *et al.* (2012 ▸) do not report on any hydrogen bonding within the structure of **2** but the C—H⋯S distances are very similar than those in Table 1 ▸.

## Synthesis and crystallization   

Tris-(2,2,6,6-tetra­methyl­benzo[1,2-d;4,5-d]bis­[1,3]di­thiol-4-yl)methanol **2** was obtained following the procedure of Jassoy *et al.* (2017 ▸). The synthesis of the title compound **1** was reported in the literature (Karlson *et al.*, 2014 ▸). However, the procedure was changed slightly, resulting in a more convenient work-up and increased yield.

Tris-(2,2,6,6-tetra­methyl­benzo[1,2-*d*;4,5-d]bis­[1,3]di­thiol-4-yl)methanol **2** (4.00 g, 4.52 mmol) was dissolved in 200 mL of dry diethyl ether under argon. Dry tetra­methyl­ethylendi­amine (6.80 mL, 5.24 g, 45.1 mmol, 10 eq.) was added and the solution was cooled to 273 K. Subsequently, *n*-butyl lithium (2.5 *M* in hexa­nes, 18.08 mL, 45.2 mmol, 10 eq.) was added dropwise. The reaction mixture was allowed to warm up to room temperature while stirring for 3 h. Afterwards, the reaction mixture was cooled down to 195 K and tri­methyl­silyl chloride (6.30 mL, 5.40 g, 49.7 mmol, 11.0 eq.) was added dropwise. Then, the cooling bath was removed and the mixture was stirred for 16 h at room temperature. The reaction was then quenched with 10 mL 1 *M* NaOH and the organic solvents were removed under reduced pressure. The dark-greenish residue was taken up in methyl­ene chloride (200 mL) and washed with water (200 mL) twice. The organic phase was separated and dried over sodium sulfate. After removal of the solvents under reduced pressure, the crude product was purified by washing with acetone. For that, the residue was suspended in acetone (50 mL) and treated with ultrasound for 3 min. Then, the mixture was centrifuged at 3200 g (Eppendorf Centrifuge 5810 R) for 5 min, whereupon a colorless solid separated. This procedure was repeated with the precipitated solid three times, until the supernatant was clear and almost colorless. The pure product was obtained as a colorless solid after drying the precipitate under vacuum with a yield of 3.32g (3.01 mmol, 67%). The pure product was then crystallized in the following way: compound **1** was dissolved in diethyl ether, the clear solution placed in an open tube at 278 K and the solvent was slowly evaporated over three days. This yielded light-yellow plates of **1** suitable for X-ray diffraction.

^1^H NMR (500 MHz, CD_2_Cl_2_, 298 K, δ in ppm): 6.50 (*s*, 1H), 1.77 (*s*, 18H), 1.65 (*s*, 9H), 1.61 (*s*, 9H), 0.46 (*s*, 27H). ^13^C NMR (126 MHz, CD_2_Cl_2_, 298 K, δ in ppm): 144.92, 144.53, 140.83, 138.79, 133.56, 130.66, 85.11, 62.13, 61.86, 34.92, 32.24, 29.33, 27.20, 2.66. The assignment of NMR signals for trityl alcohols has been discussed by Tormyshev *et al.* (2012 ▸). ESI(+) (*m*/*z*): 1100.089 [*M*]^+^, 1123.078 [*M* + Na]^+^. HRMS–ESI(+): 1100.0908 (calculated for C_46_H_64_OS_12_Si_3_: 1100.0908). Elemental analysis [%]: C 49.33, H 5.77, S 33.95 (calculated for C_46_H_64_OS_12_Si_3_: C 50.14, H 5.85, S 34.91).

## Refinement   

Crystal data, data collection and structure refinement details are summarized in Table 2 ▸. H atoms were positioned geometrically and refined using a riding model as idealized hy­droxy and methyl groups (SHELXL AFIX codes 147 and 137), thus including free rotation around the respective C—O and C—C bonds. *U*_iso_(H) was set to 1.5 times *U*_eq_(C,O). At a first attempt, a diethyl ether solvent mol­ecule was modeled over three partially occupied positions summing up to one mol­ecule. This model still contained a residual of approximately two electrons, which could not be further incorporated into an appropriate model of a fourth orientation of the ether. Therefore, we decided to use the *PLATON* SQUEEZE (Spek, 2015 ▸) solvent masking procedure as implemented in *OLEX2* (Dolomanov *et al.*, 2009 ▸). The calculated solvent void in the unit cell has a volume of 580 Å^3^ and 127 e have been recovered. The previous model of the refined parts of the diethyl ether mol­ecules without the use of solvent masking is added as a part of a *SHELXL* res file to the refine_special_details section of the CIF file. The C5-bonded tri­methyl­silyl group shows a half-to-half disorder over two positions slightly above and below the plane of the respective phenyl ring. This disorder could be resolved by individual refinement of the respective parts with occupancy factors linked together *via* a free variable [occupancy ratio 0.504 (4):0.496 (4)]. Additionally two Si—C distance restraints to 1.80 (1) Å were applied for two Si—C bonds, and some *U*_iso_ and *U*_aniso_ restraints were used. Atom S2 is disordered over two positions in a 0.509 (7):0.491 (7) ratio. The two disordered S atoms were treated with SIMU/ISOR restraints; the bond lengths to neighbouring atoms C4 and C8 were subjected to a SADI restraint.

## Supplementary Material

Crystal structure: contains datablock(s) I, global. DOI: 10.1107/S2056989018004516/nk2246sup1.cif

Structure factors: contains datablock(s) I. DOI: 10.1107/S2056989018004516/nk2246Isup2.hkl

CCDC reference: 1829596

Additional supporting information:  crystallographic information; 3D view; checkCIF report

## Figures and Tables

**Table 1:** Hydrogen-bond geometry (Å, °)

*D*—H⋯*A*	*D*—H	H⋯*A*	*D*⋯*A*	*D*—H⋯*A*
O1—H1⋯S8	0.84	2.32	3.031 (2)	142
C9—H9*C*⋯S5	0.98	3.05	3.926 (5)	150
C12—H12*A*⋯S3	0.98	2.51	3.184 (16)	126
C13—H13*B*⋯S6^i^	0.98	2.85	3.734 (10)	150
C15—H15*B*⋯S11^ii^	0.98	3.00	3.866 (4)	148
C16—H16*C*⋯S5	0.98	3.00	3.912 (4)	155
C26—H26*C*⋯S6	0.98	2.68	3.364 (5)	127
C31—H31*A*⋯S12^ii^	0.98	2.81	3.435 (3)	123
C41—H41*A*⋯S10	0.98	2.87	3.508 (5)	123
C42—H42*C*⋯S10	0.98	2.60	3.291 (3)	128
C45—H45*C*⋯S1	0.98	2.96	3.867 (4)	155

Symmetry codes: (i) 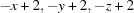; (ii) 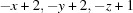.

**Table 2:** Experimental details

Crystal data	
Chemical formula	C_46_H_64_OS_12_Si_3_
*M*_r_	1101.96
Crystal system, space group	Triclinic, *P* 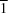
Temperature (K)	100
*a*, *b*, *c* (Å)	14.9964 (4), 15.1070 (4), 16.0026 (4)
α, β, γ (°)	91.6815 (13), 117.6083 (11), 99.1383 (12)
*V* (Å^3^)	3149.79 (15)
*Z*	2
Radiation type	Cu *K*α
μ (mm^−1^)	4.64
Crystal size (mm)	0.34 × 0.18 × 0.04

Data collection	
Diffractometer	Bruker D8-Venture
Absorption correction	Multi-scan (*SADABS*; Bruker, 2015 ▸)
*T*_min_, *T*_max_	0.252, 0.754
No. of measured, independent and observed [*I* > 2σ(*I*)] reflections	74526, 11369, 9723
*R*_int_	0.091
(sin θ/λ)_max_ (Å^−1^)	0.600

Refinement	
*R*[*F* ^2^ > 2σ(*F* ^2^)], *wR*(*F* ^2^), *S*	0.064, 0.183, 1.03
No. of reflections	11369
No. of parameters	632
No. of restraints	214
H-atom treatment	H-atom parameters constrained
Δρ_max_, Δρ_min_ (e Å^−3^)	0.89, −1.34

Computer programs: *SMART* and *SAINT* (Bruker, 2015 ▸), *SHELXS* (Sheldrick, 2008 ▸), *SHELXL2014/7* (Sheldrick, 2015 ▸) and *OLEX2* (Dolomanov *et al.*, 2009 ▸).

## supplementary crystallographic information

### Crystal data

**Table:**

C_46_H_64_OS_12_Si_3_	*Z* = 2
*M**_r_* = 1101.96	*F*(000) = 1164
Triclinic, *P*1	*D*_x_ = 1.162 Mg m^−^^3^
*a* = 14.9964 (4) Å	Cu *K*α radiation, λ = 1.54178 Å
*b* = 15.1070 (4) Å	Cell parameters from 9511 reflections
*c* = 16.0026 (4) Å	θ = 3.0–72.3°
α = 91.6815 (13)°	µ = 4.64 mm^−^^1^
β = 117.6083 (11)°	*T* = 100 K
γ = 99.1383 (12)°	Plate, clear yellowish blue
*V* = 3149.79 (15) Å^3^	0.34 × 0.18 × 0.04 mm

### Data collection

**Table:**

Bruker D8-Venture diffractometer	11369 independent reflections
Radiation source: microfocus sealed X-ray tube, Incoatec Iµs	9723 reflections with *I* > 2σ(*I*)
Mirror optics monochromator	*R*_int_ = 0.091
Detector resolution: 7.9 pixels mm^-1^	θ_max_ = 67.7°, θ_min_ = 3.0°
fine slicing ω and φ scans	*h* = −18→17
Absorption correction: multi-scan (SADABS; Bruker, 2015)	*k* = −18→18
*T*_min_ = 0.252, *T*_max_ = 0.754	*l* = −19→19
74526 measured reflections	

### Refinement

**Table:**

Refinement on *F*^2^	Hydrogen site location: inferred from neighbouring sites
Least-squares matrix: full	H-atom parameters constrained
*R*[*F*^2^ > 2σ(*F*^2^)] = 0.064	*w* = 1/[σ^2^(*F*_o_^2^) + (0.1335*P*)^2^ + 2.6751*P*] where *P* = (*F*_o_^2^ + 2*F*_c_^2^)/3
*wR*(*F*^2^) = 0.183	(Δ/σ)_max_ = 0.001
*S* = 1.03	Δρ_max_ = 0.89 e Å^−^^3^
11369 reflections	Δρ_min_ = −1.34 e Å^−^^3^
632 parameters	Extinction correction: *SHELXL2014/7* (Sheldrick 2015), Fc^*^=kFc[1+0.001xFc^2^λ^3^/sin(2θ)]^-1/4^
214 restraints	Extinction coefficient: 0.0018 (2)
Primary atom site location: structure-invariant direct methods	

### Special details

**Table:**

Geometry. All esds (except the esd in the dihedral angle between two l.s. planes) are estimated using the full covariance matrix. The cell esds are taken into account individually in the estimation of esds in distances, angles and torsion angles; correlations between esds in cell parameters are only used when they are defined by crystal symmetry. An approximate (isotropic) treatment of cell esds is used for estimating esds involving l.s. planes.
Refinement. H atoms were positioned geometrically and refined using a riding model as idealised hydroxy- and methyl groups (AFIX codes 147 and 137), thus including free rotation around the respective C-O and C-C bonds. The *U*_iso_(H) was set to 1.5 times *U*_eq_(C/O). At a first attempt a diethyl ether solvent molecule was modeled over three partially occupied positions summing up to one molecule. This model contained still Q-peaks of approx. 2 electrons, which could no be further incorporated into an appropriate model of a forth orientation of the ether. Therefore, we decided to use the solvent masking procedure - as implemented in Olex2 (Dolomanov *et al.*, 2009)). The previous model of the refined parts of diethyl ether molecules is added as a part of a Shelx-RES-file to this section. The C5-bonded trimethylsilyl group shows a half-to-half disorder over two positions slightly above and below the plane of the respective phenyl ring. This disorder could be resolved by individual refinement of the respective parts with occupancy factors linked together via a free variable. Additionally two Si-C distance restraints to 180 (1) pm has been applied for two Si-C bonds, and some *U*_iso_ and *U*_aniso_ restraints were used.PART OF THE RES-FILE FOR THE DISORDERED DIETHYL ETHER MOLECULE INCLUDING Q-PEAKS: >>> DFIX 1.48 0.01 O2 C49 O2S C49S DFIX 2.4 0.01 O2 C50 O2 C47 O2S C50S O2S C47S DFIX 1.48 0.01 O2 C48 O2S C48S DFIX 2.4 0.01 O2 C47 O2S C47S DFIX 1.54 0.01 C50 C49 C48 C47 C50S C49S C48S C47S DFIX 1.54 0.01 C50T C49T DFIX 1.54 0.01 C47T C48T DFIX 1.48 0.01 C48T O2T DFIX 1.48 0.01 O2T C49T DFIX 2.48 0.01 C48T C49T DFIX 2.48 0.01 C47T O2T DFIX 2.48 0.01 O2T C50T SIMU 0.01 0.02 2 O2T > C50T SIMU 0.01 0.02 2 O2S > C50S SIMU 0.01 0.02 2 O2 > C50 RIGU 0.01 0.01 O2 > C50S RIGU 0.01 0.01 O2 > C50T ISOR 0.02 0.04 C50S C47T C47S C48S O2S C49S C50T C49T O2T C48T C47 C48 O2 = C49 C50 ISOR 0.01 0.02 C50S C47T ISOR 0.005 0.01 C47T C50S ISOR 0.001 0.002 C50S ISOR 0.005 0.01 C48 C47T ISOR 0.005 0.01 C50 SUMP 1 0.001 1 3 1 4 1 5FVAR 0.13459 0.50735 0.24003 0.3846 0.3772 PART 1 O2 O 0.79116 0.65541 0.99956 31.00000 0.06543 0.04975 0.04965 = -0.00072 0.04561 0.00256 C47 C 0.68695 0.76136 0.91836 31.00000 0.03672 0.06954 0.04613 = -0.00159 0.02800 0.00548 AFIX 33 H47A H 0.62092 0.77987 0.89833 31.00000 -1.50000 H47B H 0.74274 0.81100 0.95993 31.00000 -1.50000 H47C H 0.69454 0.74611 0.86234 31.00000 -1.50000 AFIX 0 C48 C 0.69114 0.67801 0.97272 31.00000 0.04645 0.04377 0.04303 = 0.00343 0.02874 -0.00366 AFIX 23 H48A H 0.68391 0.69227 1.02980 31.00000 -1.20000 H48B H 0.63544 0.62702 0.93166 31.00000 -1.20000 AFIX 0 C49 C 0.81142 0.57840 1.05476 31.00000 0.07746 0.04791 0.05614 = 0.00453 0.03660 -0.00465 AFIX 23 H49A H 0.76026 0.52318 1.01798 31.00000 -1.20000 H49B H 0.80914 0.59064 1.11479 31.00000 -1.20000 AFIX 0 C50 C 0.92022 0.56745 1.07495 31.00000 0.07775 0.03830 0.02915 = 0.00545 0.03727 0.02086 AFIX 33 H50A H 0.93907 0.51666 1.11200 31.00000 -1.50000 H50B H 0.92097 0.55586 1.01472 31.00000 -1.50000 H50C H 0.96954 0.62290 1.11092 31.00000 -1.50000 AFIX 0 PART 0 PART 2 O2S O 0.80496 0.41812 0.83113 51.00000 0.04242 0.01173 0.02512 = 0.00026 0.02239 0.00148 C47S C 0.63092 0.43229 0.78311 51.00000 0.05496 0.02781 0.10646 = 0.00791 0.05577 0.02148 AFIX 33 H47D H 0.55907 0.40093 0.74948 51.00000 -1.50000 H47E H 0.64970 0.45113 0.84923 51.00000 -1.50000 H47F H 0.63991 0.48557 0.75225 51.00000 -1.50000 AFIX 0 C48S C 0.69956 0.36881 0.78057 51.00000 0.04342 0.01560 0.04336 = 0.00295 0.02662 0.01208 AFIX 23 H48C H 0.69101 0.31461 0.81144 51.00000 -1.20000 H48D H 0.68117 0.34919 0.71407 51.00000 -1.20000 AFIX 0 C49S C 0.87446 0.36079 0.83049 51.00000 0.03885 0.00950 0.01506 = 0.00470 0.01092 -0.00216 AFIX 23 H49C H 0.85738 0.34134 0.76431 51.00000 -1.20000 H49D H 0.86760 0.30627 0.86155 51.00000 -1.20000 AFIX 0 C50S C 0.98333 0.41458 0.88367 51.00000 0.02572 0.02320 0.01865 = 0.00666 0.01154 0.00333 AFIX 33 H50D H 1.03110 0.37707 0.88395 51.00000 -1.50000 H50E H 0.98952 0.46819 0.85226 51.00000 -1.50000 H50F H 0.99971 0.43324 0.94917 51.00000 -1.50000 AFIX 0 PART 0 PART 3 O2T O 0.68349 0.55785 0.87942 41.00000 0.08189 0.06899 0.08683 = 0.01661 0.05060 0.01595 C47T C 0.69930 0.70010 0.96924 41.00000 0.03297 0.06769 0.07028 = -0.01037 0.03848 -0.00697 AFIX 33 H47G H 0.73596 0.73543 1.03204 41.00000 -1.50000 H47H H 0.71121 0.73487 0.92339 41.00000 -1.50000 H47I H 0.62566 0.68654 0.94920 41.00000 -1.50000 AFIX 0 C48T C 0.73819 0.61238 0.97402 41.00000 0.06026 0.06820 0.08184 = 0.01147 0.04640 -0.00876 AFIX 23 H48E H 0.81276 0.62594 0.99507 41.00000 -1.20000 H48F H 0.72706 0.57752 1.02093 41.00000 -1.20000 AFIX 0 C49T C 0.72052 0.47357 0.87752 41.00000 0.08416 0.05573 0.09867 = 0.03191 0.04896 0.01075 AFIX 23 H49E H 0.72175 0.44039 0.93037 41.00000 -1.20000 H49F H 0.79146 0.48813 0.88675 41.00000 -1.20000 AFIX 0 C50T C 0.65152 0.41355 0.78303 41.00000 0.10387 0.04811 0.11528 = 0.03104 0.06179 0.01396 AFIX 33 H50G H 0.67780 0.35804 0.78363 41.00000 -1.50000 H50H H 0.58152 0.39827 0.77432 41.00000 -1.50000 H50I H 0.65115 0.44596 0.73075 41.00000 -1.50000 AFIX 0 PART 0 Q1 Q 0.87840 0.64330 1.06050 11.00000 0.05000 2.190 Q2 Q 0.94500 0.37550 0.85740 11.00000 0.05000 1.270 <<<

### Fractional atomic coordinates and isotropic or equivalent isotropic displacement parameters (Å^2^)

**Table:**

	*x*	*y*	*z*	*U*_iso_*/*U*_eq_	Occ. (<1)
S1	0.69743 (6)	0.71217 (5)	0.52986 (6)	0.01790 (19)	
S2S	0.5696 (2)	0.81471 (17)	0.5664 (3)	0.0154 (5)	0.509 (7)
S3	0.89076 (6)	1.11185 (6)	0.72696 (6)	0.0236 (2)	
S4	1.00639 (5)	1.02542 (5)	0.65703 (5)	0.01273 (18)	
S5	0.93657 (6)	0.81063 (6)	0.77883 (5)	0.0213 (2)	
S6	1.13719 (7)	0.82128 (7)	0.93714 (6)	0.0282 (2)	
S7	1.30244 (6)	0.79203 (5)	0.69151 (6)	0.01820 (19)	
S8	1.10059 (5)	0.77389 (5)	0.53140 (5)	0.01330 (18)	
S9	0.95695 (6)	0.61647 (5)	0.61759 (5)	0.01480 (19)	
S10	0.84595 (6)	0.47280 (5)	0.46182 (5)	0.01626 (19)	
S11	0.65178 (6)	0.71730 (5)	0.21722 (5)	0.01615 (19)	
S12	0.77538 (5)	0.86504 (5)	0.36777 (5)	0.01236 (18)	
Si1	0.69340 (15)	0.99991 (13)	0.74868 (15)	0.0252 (7)	0.504 (4)
Si1S	0.63902 (16)	1.02339 (14)	0.67283 (16)	0.0270 (7)	0.496 (4)
Si2	1.34975 (7)	0.82358 (6)	0.92788 (6)	0.0223 (2)	
Si3	0.66839 (6)	0.49178 (6)	0.24913 (6)	0.0150 (2)	
O1	0.95362 (15)	0.89480 (13)	0.52132 (14)	0.0102 (4)	
H1	0.9979	0.8807	0.5080	0.015*	
C1	0.9234 (2)	0.82424 (19)	0.5669 (2)	0.0106 (4)	
C2	0.8534 (2)	0.86769 (19)	0.5959 (2)	0.0107 (5)	
C3	0.7569 (2)	0.8247 (2)	0.5815 (2)	0.0145 (6)	
C4	0.7001 (3)	0.8704 (2)	0.6112 (3)	0.0233 (7)	
C5	0.7365 (3)	0.9575 (3)	0.6598 (3)	0.0323 (9)	
C6	0.8325 (3)	0.9997 (2)	0.6714 (2)	0.0213 (7)	
C7	0.8895 (2)	0.9575 (2)	0.6391 (2)	0.0124 (6)	
C8	0.6076 (3)	0.6992 (3)	0.5781 (3)	0.0299 (8)	
C9	0.6567 (4)	0.6697 (4)	0.6763 (4)	0.0552 (12)	
H9A	0.6771	0.6117	0.6730	0.083*	
H9B	0.6075	0.6631	0.7010	0.083*	
H9C	0.7174	0.7151	0.7185	0.083*	
C10	0.5117 (3)	0.6320 (3)	0.5099 (3)	0.0356 (9)	
H10A	0.4805	0.6545	0.4478	0.053*	
H10B	0.4627	0.6242	0.5348	0.053*	
H10C	0.5300	0.5738	0.5029	0.053*	
C11	0.5648 (8)	0.9405 (7)	0.7318 (8)	0.035 (3)	0.504 (4)
H11A	0.5128	0.9394	0.6656	0.053*	0.504 (4)
H11B	0.5462	0.9727	0.7736	0.053*	0.504 (4)
H11C	0.5686	0.8785	0.7474	0.053*	0.504 (4)
C11S	0.6114 (11)	0.9658 (9)	0.7616 (9)	0.048 (3)	0.496 (4)
H11D	0.5878	0.9009	0.7404	0.072*	0.496 (4)
H11E	0.5580	0.9903	0.7681	0.072*	0.496 (4)
H11F	0.6740	0.9758	0.8230	0.072*	0.496 (4)
C12	0.6858 (10)	1.1186 (6)	0.7389 (9)	0.050 (3)	0.504 (4)
H12A	0.7547	1.1546	0.7612	0.075*	0.504 (4)
H12B	0.6563	1.1385	0.7778	0.075*	0.504 (4)
H12C	0.6422	1.1264	0.6724	0.075*	0.504 (4)
C12S	0.6782 (9)	1.1423 (8)	0.7090 (8)	0.042 (3)	0.496 (4)
H12D	0.7322	1.1536	0.7753	0.063*	0.496 (4)
H12E	0.6193	1.1674	0.7028	0.063*	0.496 (4)
H12F	0.7045	1.1712	0.6687	0.063*	0.496 (4)
C13	0.7901 (7)	0.9908 (8)	0.8727 (6)	0.053 (3)	0.504 (4)
H13A	0.7800	0.9283	0.8865	0.080*	0.504 (4)
H13B	0.7819	1.0307	0.9170	0.080*	0.504 (4)
H13C	0.8592	1.0086	0.8798	0.080*	0.504 (4)
C13S	0.5219 (5)	1.0181 (6)	0.5587 (5)	0.037 (2)	0.496 (4)
H13D	0.5409	1.0336	0.5093	0.056*	0.496 (4)
H13E	0.4820	1.0609	0.5647	0.056*	0.496 (4)
H13F	0.4806	0.9568	0.5414	0.056*	0.496 (4)
C14	1.0211 (3)	1.1019 (2)	0.7552 (2)	0.0200 (7)	
C15	1.0781 (3)	1.1956 (2)	0.7551 (3)	0.0259 (8)	
H15A	1.0451	1.2145	0.6914	0.039*	
H15B	1.1496	1.1931	0.7732	0.039*	
H15C	1.0761	1.2390	0.8006	0.039*	
C16	1.0746 (3)	1.0643 (3)	0.8492 (2)	0.0279 (8)	
H16A	1.0820	1.1065	0.9007	0.042*	
H16B	1.1425	1.0560	0.8603	0.042*	
H16C	1.0337	1.0060	0.8472	0.042*	
C17	1.0223 (2)	0.80852 (19)	0.6547 (2)	0.0122 (5)	
C18	1.0354 (2)	0.8110 (2)	0.7474 (2)	0.0145 (6)	
C19	1.1330 (3)	0.8122 (2)	0.8245 (2)	0.0189 (7)	
C20	1.2209 (2)	0.8106 (2)	0.8155 (2)	0.0180 (7)	
C21	1.2046 (2)	0.8029 (2)	0.7213 (2)	0.0142 (6)	
C22	1.1077 (2)	0.79875 (19)	0.6436 (2)	0.0125 (6)	
C23	1.0042 (3)	0.7670 (3)	0.8926 (2)	0.0288 (8)	
C24	0.9651 (3)	0.7987 (3)	0.9588 (3)	0.0409 (11)	
H24A	0.9763	0.8648	0.9660	0.061*	
H24B	1.0023	0.7780	1.0210	0.061*	
H24C	0.8916	0.7738	0.9318	0.061*	
C25	0.9910 (4)	0.6646 (3)	0.8790 (3)	0.0429 (11)	
H25A	0.9186	0.6370	0.8552	0.064*	
H25B	1.0326	0.6434	0.9399	0.064*	
H25C	1.0133	0.6475	0.8331	0.064*	
C26	1.3716 (3)	0.9374 (3)	0.9911 (3)	0.0285 (8)	
H26A	1.3768	0.9841	0.9515	0.043*	
H26B	1.4354	0.9466	1.0514	0.043*	
H26C	1.3140	0.9412	1.0034	0.043*	
C27	1.3473 (4)	0.7314 (3)	1.0037 (3)	0.0403 (11)	
H27A	1.2910	0.6809	0.9650	0.060*	
H27B	1.3371	0.7548	1.0558	0.060*	
H27C	1.4126	0.7104	1.0300	0.060*	
C28	1.4621 (3)	0.8195 (3)	0.9072 (3)	0.0347 (9)	
H28A	1.4504	0.7620	0.8702	0.052*	
H28B	1.5243	0.8248	0.9684	0.052*	
H28C	1.4704	0.8695	0.8721	0.052*	
C29	1.2354 (2)	0.8224 (2)	0.5714 (2)	0.0154 (6)	
C30	1.2719 (3)	0.7776 (2)	0.5092 (3)	0.0228 (7)	
H30A	1.3445	0.8029	0.5313	0.034*	
H30B	1.2314	0.7886	0.4432	0.034*	
H30C	1.2631	0.7124	0.5132	0.034*	
C31	1.2502 (3)	0.9242 (2)	0.5710 (3)	0.0214 (7)	
H31A	1.2271	0.9503	0.6126	0.032*	
H31B	1.2100	0.9385	0.5061	0.032*	
H31C	1.3230	0.9495	0.5939	0.032*	
C32	0.8655 (2)	0.74079 (19)	0.4918 (2)	0.0109 (4)	
C33	0.8767 (2)	0.6516 (2)	0.5076 (2)	0.0115 (4)	
C34	0.8192 (2)	0.5809 (2)	0.4325 (2)	0.0120 (5)	
C35	0.7463 (2)	0.5947 (2)	0.3412 (2)	0.0141 (6)	
C36	0.7371 (2)	0.6845 (2)	0.3271 (2)	0.0119 (5)	
C37	0.7959 (2)	0.7566 (2)	0.3995 (2)	0.0111 (4)	
C38	0.8890 (2)	0.4986 (2)	0.5879 (2)	0.0166 (7)	
C39	0.9658 (3)	0.4392 (2)	0.6430 (2)	0.0203 (7)	
H39A	1.0200	0.4457	0.6244	0.030*	
H39B	0.9303	0.3760	0.6285	0.030*	
H39C	0.9961	0.4578	0.7112	0.030*	
C40	0.7995 (3)	0.4882 (2)	0.6093 (2)	0.0215 (7)	
H40A	0.8251	0.5054	0.6772	0.032*	
H40B	0.7631	0.4251	0.5921	0.032*	
H40C	0.7524	0.5272	0.5724	0.032*	
C41	0.5858 (3)	0.4220 (2)	0.2915 (3)	0.0235 (7)	
H41A	0.6292	0.4044	0.3540	0.035*	
H41B	0.5462	0.3677	0.2463	0.035*	
H41C	0.5386	0.4573	0.2965	0.035*	
C42	0.7586 (3)	0.4307 (2)	0.2315 (2)	0.0222 (7)	
H42A	0.7983	0.4708	0.2084	0.033*	
H42B	0.7194	0.3773	0.1848	0.033*	
H42C	0.8054	0.4118	0.2920	0.033*	
C43	0.5797 (3)	0.5163 (2)	0.1276 (2)	0.0269 (8)	
H43A	0.5279	0.5466	0.1304	0.040*	
H43B	0.5458	0.4596	0.0851	0.040*	
H43C	0.6189	0.5555	0.1034	0.040*	
C44	0.6490 (2)	0.8268 (2)	0.2665 (2)	0.0177 (7)	
C45	0.5651 (3)	0.8175 (3)	0.2959 (3)	0.0254 (8)	
H45A	0.4984	0.7943	0.2403	0.038*	
H45B	0.5647	0.8766	0.3224	0.038*	
H45C	0.5782	0.7754	0.3439	0.038*	
C46	0.6336 (3)	0.8933 (2)	0.1934 (2)	0.0250 (8)	
H46A	0.6883	0.8972	0.1756	0.038*	
H46B	0.6356	0.9530	0.2206	0.038*	
H46C	0.5670	0.8726	0.1369	0.038*	
S2	0.5825 (2)	0.80229 (18)	0.5960 (3)	0.0152 (5)	0.491 (7)

### Atomic displacement parameters (Å^2^)

**Table:**

	*U*^11^	*U*^22^	*U*^33^	*U*^12^	*U*^13^	*U*^23^
S1	0.0118 (4)	0.0104 (4)	0.0299 (4)	−0.0058 (3)	0.0113 (3)	−0.0037 (3)
S2S	0.0108 (6)	0.0183 (7)	0.0175 (8)	−0.0042 (5)	0.0095 (6)	−0.0011 (6)
S3	0.0157 (4)	0.0176 (4)	0.0356 (4)	−0.0051 (3)	0.0142 (3)	−0.0159 (3)
S4	0.0086 (3)	0.0088 (3)	0.0184 (4)	−0.0047 (3)	0.0069 (3)	−0.0069 (3)
S5	0.0151 (4)	0.0295 (5)	0.0164 (4)	−0.0050 (3)	0.0080 (3)	−0.0001 (3)
S6	0.0222 (5)	0.0387 (5)	0.0134 (4)	−0.0039 (4)	0.0032 (3)	−0.0009 (3)
S7	0.0081 (3)	0.0157 (4)	0.0254 (4)	0.0018 (3)	0.0038 (3)	−0.0004 (3)
S8	0.0071 (3)	0.0111 (4)	0.0178 (4)	−0.0021 (3)	0.0043 (3)	−0.0056 (3)
S9	0.0125 (4)	0.0075 (3)	0.0162 (3)	−0.0028 (3)	0.0017 (3)	−0.0010 (3)
S10	0.0159 (4)	0.0058 (4)	0.0190 (4)	−0.0024 (3)	0.0033 (3)	−0.0034 (3)
S11	0.0121 (4)	0.0112 (4)	0.0154 (4)	−0.0008 (3)	−0.0003 (3)	−0.0036 (3)
S12	0.0080 (3)	0.0065 (3)	0.0163 (4)	−0.0018 (3)	0.0018 (3)	−0.0026 (3)
Si1	0.0163 (10)	0.0207 (11)	0.0404 (14)	−0.0054 (8)	0.0189 (10)	−0.0148 (8)
Si1S	0.0232 (12)	0.0224 (11)	0.0444 (15)	0.0027 (8)	0.0246 (11)	−0.0049 (9)
Si2	0.0154 (5)	0.0156 (5)	0.0202 (5)	0.0001 (3)	−0.0033 (4)	−0.0038 (3)
Si3	0.0111 (4)	0.0091 (4)	0.0173 (4)	−0.0048 (3)	0.0032 (3)	−0.0061 (3)
O1	0.0076 (8)	0.0078 (8)	0.0149 (8)	−0.0022 (7)	0.0065 (7)	−0.0021 (7)
C1	0.0079 (7)	0.0064 (7)	0.0148 (7)	−0.0021 (6)	0.0043 (6)	−0.0016 (6)
C2	0.0079 (9)	0.0072 (9)	0.0144 (9)	−0.0016 (8)	0.0043 (8)	−0.0020 (8)
C3	0.0073 (14)	0.0165 (16)	0.0173 (15)	−0.0024 (12)	0.0058 (12)	−0.0052 (12)
C4	0.0159 (13)	0.0236 (14)	0.0304 (14)	−0.0040 (11)	0.0139 (11)	−0.0077 (11)
C5	0.0251 (15)	0.0315 (16)	0.0441 (16)	0.0016 (12)	0.0218 (13)	−0.0136 (12)
C6	0.0137 (16)	0.0179 (17)	0.0291 (18)	−0.0061 (13)	0.0112 (14)	−0.0142 (13)
C7	0.0087 (12)	0.0122 (12)	0.0143 (12)	−0.0032 (10)	0.0057 (10)	−0.0014 (10)
C8	0.0224 (13)	0.0324 (14)	0.0371 (14)	−0.0026 (11)	0.0183 (11)	0.0054 (11)
C9	0.0381 (19)	0.072 (2)	0.052 (2)	−0.0080 (18)	0.0231 (17)	0.0236 (18)
C10	0.0232 (16)	0.0264 (17)	0.0545 (19)	−0.0067 (14)	0.0204 (15)	−0.0026 (15)
C11	0.031 (6)	0.029 (5)	0.052 (7)	−0.010 (4)	0.031 (5)	−0.022 (4)
C11S	0.056 (9)	0.056 (8)	0.048 (7)	−0.002 (6)	0.043 (7)	−0.006 (6)
C12	0.047 (4)	0.051 (5)	0.059 (4)	0.017 (3)	0.029 (3)	0.004 (3)
C12S	0.040 (4)	0.051 (4)	0.049 (4)	0.023 (3)	0.027 (3)	0.004 (3)
C13	0.040 (5)	0.083 (8)	0.034 (5)	−0.009 (5)	0.024 (4)	−0.027 (5)
C13S	0.027 (4)	0.027 (4)	0.064 (6)	0.015 (3)	0.024 (4)	0.009 (4)
C14	0.0133 (16)	0.0178 (17)	0.0255 (17)	−0.0057 (13)	0.0100 (14)	−0.0141 (13)
C15	0.0188 (16)	0.0171 (16)	0.0373 (18)	−0.0083 (13)	0.0144 (14)	−0.0158 (13)
C16	0.0200 (18)	0.035 (2)	0.0206 (17)	−0.0062 (15)	0.0075 (15)	−0.0136 (15)
C17	0.0095 (9)	0.0066 (9)	0.0152 (9)	−0.0028 (7)	0.0028 (7)	−0.0013 (7)
C18	0.0121 (12)	0.0114 (12)	0.0161 (12)	−0.0030 (10)	0.0052 (10)	−0.0019 (10)
C19	0.0173 (16)	0.0148 (16)	0.0173 (15)	−0.0035 (13)	0.0045 (13)	−0.0015 (12)
C20	0.0127 (15)	0.0094 (15)	0.0206 (16)	−0.0024 (12)	0.0001 (13)	−0.0020 (12)
C21	0.0106 (12)	0.0087 (12)	0.0191 (12)	−0.0012 (10)	0.0047 (10)	−0.0013 (10)
C22	0.0112 (12)	0.0068 (12)	0.0151 (12)	−0.0029 (10)	0.0044 (10)	−0.0027 (9)
C23	0.027 (2)	0.036 (2)	0.0178 (17)	−0.0071 (16)	0.0092 (15)	0.0050 (15)
C24	0.035 (2)	0.063 (3)	0.0242 (19)	−0.004 (2)	0.0181 (18)	0.0033 (18)
C25	0.051 (3)	0.039 (3)	0.031 (2)	−0.008 (2)	0.018 (2)	0.0089 (18)
C26	0.0201 (18)	0.0244 (19)	0.0277 (18)	−0.0022 (15)	0.0029 (15)	−0.0068 (14)
C27	0.041 (2)	0.025 (2)	0.030 (2)	0.0001 (18)	−0.0026 (18)	0.0044 (16)
C28	0.0200 (17)	0.039 (2)	0.0280 (18)	0.0076 (15)	−0.0027 (14)	−0.0098 (15)
C29	0.0084 (12)	0.0133 (13)	0.0215 (13)	−0.0003 (10)	0.0057 (10)	−0.0026 (10)
C30	0.0139 (16)	0.0190 (17)	0.0358 (19)	0.0009 (13)	0.0132 (15)	−0.0058 (14)
C31	0.0152 (16)	0.0127 (16)	0.0319 (18)	−0.0037 (13)	0.0096 (14)	−0.0009 (13)
C32	0.0071 (7)	0.0073 (7)	0.0153 (7)	−0.0024 (6)	0.0042 (6)	−0.0020 (6)
C33	0.0070 (8)	0.0079 (9)	0.0163 (8)	−0.0017 (7)	0.0039 (7)	−0.0021 (7)
C34	0.0077 (10)	0.0078 (10)	0.0175 (10)	−0.0011 (8)	0.0044 (8)	−0.0016 (8)
C35	0.0089 (14)	0.0123 (15)	0.0175 (15)	−0.0050 (12)	0.0057 (12)	−0.0034 (12)
C36	0.0071 (10)	0.0096 (10)	0.0154 (10)	−0.0022 (8)	0.0037 (8)	−0.0021 (8)
C37	0.0066 (8)	0.0081 (9)	0.0159 (8)	−0.0016 (7)	0.0042 (7)	−0.0027 (7)
C38	0.0148 (16)	0.0083 (14)	0.0163 (15)	−0.0054 (12)	0.0015 (13)	−0.0032 (11)
C39	0.0189 (17)	0.0125 (16)	0.0234 (16)	0.0011 (13)	0.0054 (14)	0.0037 (12)
C40	0.0221 (18)	0.0134 (16)	0.0249 (17)	−0.0038 (13)	0.0103 (14)	−0.0002 (13)
C41	0.0149 (17)	0.0154 (17)	0.0346 (19)	−0.0054 (13)	0.0101 (15)	−0.0048 (14)
C42	0.0212 (17)	0.0158 (17)	0.0239 (17)	−0.0035 (13)	0.0086 (14)	−0.0075 (13)
C43	0.0258 (19)	0.0184 (18)	0.0204 (17)	−0.0023 (14)	0.0002 (15)	−0.0063 (13)
C44	0.0139 (16)	0.0103 (15)	0.0210 (16)	0.0011 (12)	0.0022 (13)	−0.0031 (12)
C45	0.0091 (15)	0.0265 (19)	0.0325 (19)	0.0022 (14)	0.0042 (14)	−0.0083 (15)
C46	0.0234 (18)	0.0144 (17)	0.0249 (18)	0.0062 (14)	0.0005 (15)	0.0006 (13)
S2	0.0110 (6)	0.0170 (7)	0.0170 (9)	−0.0038 (5)	0.0084 (6)	−0.0010 (6)

### Geometric parameters (Å, º)

**Table:**

S1—C3	1.767 (3)	C13—H13C	0.9800
S1—C8	1.827 (4)	C13S—H13D	0.9800
S2S—C4	1.786 (4)	C13S—H13E	0.9800
S2S—C8	1.908 (5)	C13S—H13F	0.9800
S3—C6	1.774 (3)	C14—C15	1.533 (5)
S3—C14	1.823 (3)	C14—C16	1.519 (5)
S4—C7	1.776 (3)	C15—H15A	0.9800
S4—C14	1.828 (3)	C15—H15B	0.9800
S5—C18	1.771 (3)	C15—H15C	0.9800
S5—C23	1.829 (4)	C16—H16A	0.9800
S6—C19	1.775 (3)	C16—H16B	0.9800
S6—C23	1.813 (4)	C16—H16C	0.9800
S7—C21	1.768 (3)	C17—C18	1.401 (4)
S7—C29	1.823 (3)	C17—C22	1.401 (4)
S8—C22	1.773 (3)	C18—C19	1.407 (5)
S8—C29	1.827 (3)	C19—C20	1.396 (5)
S9—C33	1.766 (3)	C20—C21	1.409 (5)
S9—C38	1.832 (3)	C21—C22	1.397 (4)
S10—C34	1.768 (3)	C23—C24	1.527 (6)
S10—C38	1.819 (3)	C23—C25	1.524 (6)
S11—C36	1.773 (3)	C24—H24A	0.9800
S11—C44	1.827 (3)	C24—H24B	0.9800
S12—C37	1.761 (3)	C24—H24C	0.9800
S12—C44	1.817 (3)	C25—H25A	0.9800
Si1—C5	1.945 (4)	C25—H25B	0.9800
Si1—C11	1.884 (10)	C25—H25C	0.9800
Si1—C12	1.821 (8)	C26—H26A	0.9800
Si1—C13	1.863 (10)	C26—H26B	0.9800
Si1S—C5	1.976 (4)	C26—H26C	0.9800
Si1S—C11S	1.857 (11)	C27—H27A	0.9800
Si1S—C12S	1.788 (12)	C27—H27B	0.9800
Si1S—C13S	1.844 (7)	C27—H27C	0.9800
Si2—C20	1.909 (3)	C28—H28A	0.9800
Si2—C26	1.871 (4)	C28—H28B	0.9800
Si2—C27	1.881 (4)	C28—H28C	0.9800
Si2—C28	1.871 (4)	C29—C30	1.526 (4)
Si3—C35	1.910 (3)	C29—C31	1.519 (4)
Si3—C41	1.875 (4)	C30—H30A	0.9800
Si3—C42	1.871 (4)	C30—H30B	0.9800
Si3—C43	1.868 (4)	C30—H30C	0.9800
O1—H1	0.8400	C31—H31A	0.9800
O1—C1	1.439 (3)	C31—H31B	0.9800
C1—C2	1.550 (4)	C31—H31C	0.9800
C1—C17	1.559 (4)	C32—C33	1.401 (4)
C1—C32	1.544 (4)	C32—C37	1.413 (4)
C2—C3	1.397 (4)	C33—C34	1.414 (4)
C2—C7	1.407 (4)	C34—C35	1.408 (4)
C3—C4	1.404 (5)	C35—C36	1.400 (4)
C4—C5	1.397 (5)	C36—C37	1.407 (4)
C4—S2	1.799 (4)	C38—C39	1.530 (4)
C5—C6	1.404 (5)	C38—C40	1.518 (5)
C6—C7	1.400 (5)	C39—H39A	0.9800
C8—C9	1.511 (6)	C39—H39B	0.9800
C8—C10	1.520 (5)	C39—H39C	0.9800
C8—S2	1.705 (5)	C40—H40A	0.9800
C9—H9A	0.9800	C40—H40B	0.9800
C9—H9B	0.9800	C40—H40C	0.9800
C9—H9C	0.9800	C41—H41A	0.9800
C10—H10A	0.9800	C41—H41B	0.9800
C10—H10B	0.9800	C41—H41C	0.9800
C10—H10C	0.9800	C42—H42A	0.9800
C11—H11A	0.9800	C42—H42B	0.9800
C11—H11B	0.9800	C42—H42C	0.9800
C11—H11C	0.9800	C43—H43A	0.9800
C11S—H11D	0.9800	C43—H43B	0.9800
C11S—H11E	0.9800	C43—H43C	0.9800
C11S—H11F	0.9800	C44—C45	1.523 (5)
C12—H12A	0.9800	C44—C46	1.525 (5)
C12—H12B	0.9800	C45—H45A	0.9800
C12—H12C	0.9800	C45—H45B	0.9800
C12S—H12D	0.9800	C45—H45C	0.9800
C12S—H12E	0.9800	C46—H46A	0.9800
C12S—H12F	0.9800	C46—H46B	0.9800
C13—H13A	0.9800	C46—H46C	0.9800
C13—H13B	0.9800		
			
C3—S1—C8	96.06 (16)	C17—C18—S5	125.4 (2)
C4—S2S—C8	91.3 (2)	C17—C18—C19	120.0 (3)
C6—S3—C14	96.06 (15)	C19—C18—S5	114.6 (2)
C7—S4—C14	95.31 (14)	C18—C19—S6	114.1 (3)
C18—S5—C23	95.05 (16)	C20—C19—S6	121.8 (2)
C19—S6—C23	94.64 (16)	C20—C19—C18	124.0 (3)
C21—S7—C29	95.61 (14)	C19—C20—Si2	118.8 (2)
C22—S8—C29	94.48 (14)	C19—C20—C21	114.8 (3)
C33—S9—C38	95.63 (14)	C21—C20—Si2	126.4 (3)
C34—S10—C38	95.11 (14)	C20—C21—S7	123.1 (2)
C36—S11—C44	95.94 (14)	C22—C21—S7	114.8 (2)
C37—S12—C44	95.72 (14)	C22—C21—C20	122.1 (3)
C11—Si1—C5	116.3 (3)	C17—C22—S8	123.2 (2)
C12—Si1—C5	109.0 (5)	C21—C22—S8	114.7 (2)
C12—Si1—C11	106.2 (6)	C21—C22—C17	122.0 (3)
C12—Si1—C13	107.7 (6)	S6—C23—S5	104.15 (18)
C13—Si1—C5	110.1 (3)	C24—C23—S5	108.2 (3)
C13—Si1—C11	107.2 (5)	C24—C23—S6	109.3 (3)
C11S—Si1S—C5	103.6 (5)	C25—C23—S5	111.0 (3)
C12S—Si1S—C5	118.5 (4)	C25—C23—S6	111.2 (3)
C12S—Si1S—C11S	109.7 (6)	C25—C23—C24	112.6 (3)
C12S—Si1S—C13S	101.5 (5)	C23—C24—H24A	109.5
C13S—Si1S—C5	111.8 (3)	C23—C24—H24B	109.5
C13S—Si1S—C11S	112.0 (5)	C23—C24—H24C	109.5
C26—Si2—C20	105.85 (16)	H24A—C24—H24B	109.5
C26—Si2—C27	111.40 (19)	H24A—C24—H24C	109.5
C26—Si2—C28	107.75 (18)	H24B—C24—H24C	109.5
C27—Si2—C20	110.87 (17)	C23—C25—H25A	109.5
C28—Si2—C20	115.02 (16)	C23—C25—H25B	109.5
C28—Si2—C27	106.0 (2)	C23—C25—H25C	109.5
C41—Si3—C35	107.76 (14)	H25A—C25—H25B	109.5
C42—Si3—C35	108.79 (14)	H25A—C25—H25C	109.5
C42—Si3—C41	114.03 (16)	H25B—C25—H25C	109.5
C43—Si3—C35	115.80 (15)	Si2—C26—H26A	109.5
C43—Si3—C41	106.23 (17)	Si2—C26—H26B	109.5
C43—Si3—C42	104.41 (17)	Si2—C26—H26C	109.5
C1—O1—H1	109.5	H26A—C26—H26B	109.5
O1—C1—C2	101.3 (2)	H26A—C26—H26C	109.5
O1—C1—C17	107.7 (2)	H26B—C26—H26C	109.5
O1—C1—C32	107.1 (2)	Si2—C27—H27A	109.5
C2—C1—C17	112.2 (2)	Si2—C27—H27B	109.5
C32—C1—C2	113.5 (2)	Si2—C27—H27C	109.5
C32—C1—C17	114.0 (2)	H27A—C27—H27B	109.5
C3—C2—C1	125.0 (3)	H27A—C27—H27C	109.5
C3—C2—C7	117.6 (3)	H27B—C27—H27C	109.5
C7—C2—C1	117.4 (3)	Si2—C28—H28A	109.5
C2—C3—S1	125.3 (2)	Si2—C28—H28B	109.5
C2—C3—C4	120.3 (3)	Si2—C28—H28C	109.5
C4—C3—S1	114.5 (2)	H28A—C28—H28B	109.5
C3—C4—S2S	114.0 (3)	H28A—C28—H28C	109.5
C3—C4—S2	114.4 (3)	H28B—C28—H28C	109.5
C5—C4—S2S	121.9 (3)	S7—C29—S8	104.30 (16)
C5—C4—C3	123.5 (3)	C30—C29—S7	108.9 (2)
C5—C4—S2	121.6 (3)	C30—C29—S8	109.5 (2)
C4—C5—Si1	123.0 (3)	C31—C29—S7	111.3 (2)
C4—C5—Si1S	119.0 (3)	C31—C29—S8	110.3 (2)
C4—C5—C6	114.9 (3)	C31—C29—C30	112.2 (3)
C6—C5—Si1	117.6 (3)	C29—C30—H30A	109.5
C6—C5—Si1S	123.6 (3)	C29—C30—H30B	109.5
C5—C6—S3	122.3 (3)	C29—C30—H30C	109.5
C7—C6—S3	114.6 (2)	H30A—C30—H30B	109.5
C7—C6—C5	123.1 (3)	H30A—C30—H30C	109.5
C2—C7—S4	124.3 (2)	H30B—C30—H30C	109.5
C6—C7—S4	115.3 (2)	C29—C31—H31A	109.5
C6—C7—C2	120.5 (3)	C29—C31—H31B	109.5
S1—C8—S2S	101.3 (2)	C29—C31—H31C	109.5
C9—C8—S1	110.6 (3)	H31A—C31—H31B	109.5
C9—C8—S2S	117.7 (4)	H31A—C31—H31C	109.5
C9—C8—C10	111.8 (4)	H31B—C31—H31C	109.5
C9—C8—S2	104.0 (4)	C33—C32—C1	125.4 (3)
C10—C8—S1	108.5 (3)	C33—C32—C37	117.8 (3)
C10—C8—S2S	106.1 (3)	C37—C32—C1	116.8 (3)
C10—C8—S2	112.7 (3)	C32—C33—S9	125.3 (2)
S2—C8—S1	109.2 (2)	C32—C33—C34	119.9 (3)
C8—C9—H9A	109.5	C34—C33—S9	114.7 (2)
C8—C9—H9B	109.5	C33—C34—S10	114.7 (2)
C8—C9—H9C	109.5	C35—C34—S10	122.1 (2)
H9A—C9—H9B	109.5	C35—C34—C33	123.2 (3)
H9A—C9—H9C	109.5	C34—C35—Si3	118.5 (2)
H9B—C9—H9C	109.5	C36—C35—Si3	125.8 (2)
C8—C10—H10A	109.5	C36—C35—C34	115.7 (3)
C8—C10—H10B	109.5	C35—C36—S11	123.3 (2)
C8—C10—H10C	109.5	C35—C36—C37	122.4 (3)
H10A—C10—H10B	109.5	C37—C36—S11	114.4 (2)
H10A—C10—H10C	109.5	C32—C37—S12	123.2 (2)
H10B—C10—H10C	109.5	C36—C37—S12	115.9 (2)
Si1—C11—H11A	109.5	C36—C37—C32	120.9 (3)
Si1—C11—H11B	109.5	S10—C38—S9	104.94 (16)
Si1—C11—H11C	109.5	C39—C38—S9	108.5 (2)
H11A—C11—H11B	109.5	C39—C38—S10	108.7 (2)
H11A—C11—H11C	109.5	C40—C38—S9	110.3 (2)
H11B—C11—H11C	109.5	C40—C38—S10	111.6 (2)
Si1S—C11S—H11D	109.5	C40—C38—C39	112.4 (3)
Si1S—C11S—H11E	109.5	C38—C39—H39A	109.5
Si1S—C11S—H11F	109.5	C38—C39—H39B	109.5
H11D—C11S—H11E	109.5	C38—C39—H39C	109.5
H11D—C11S—H11F	109.5	H39A—C39—H39B	109.5
H11E—C11S—H11F	109.5	H39A—C39—H39C	109.5
Si1—C12—H12A	109.5	H39B—C39—H39C	109.5
Si1—C12—H12B	109.5	C38—C40—H40A	109.5
Si1—C12—H12C	109.5	C38—C40—H40B	109.5
H12A—C12—H12B	109.5	C38—C40—H40C	109.5
H12A—C12—H12C	109.5	H40A—C40—H40B	109.5
H12B—C12—H12C	109.5	H40A—C40—H40C	109.5
Si1S—C12S—H12D	109.5	H40B—C40—H40C	109.5
Si1S—C12S—H12E	109.5	Si3—C41—H41A	109.5
Si1S—C12S—H12F	109.5	Si3—C41—H41B	109.5
H12D—C12S—H12E	109.5	Si3—C41—H41C	109.5
H12D—C12S—H12F	109.5	H41A—C41—H41B	109.5
H12E—C12S—H12F	109.5	H41A—C41—H41C	109.5
Si1—C13—H13A	109.5	H41B—C41—H41C	109.5
Si1—C13—H13B	109.5	Si3—C42—H42A	109.5
Si1—C13—H13C	109.5	Si3—C42—H42B	109.5
H13A—C13—H13B	109.5	Si3—C42—H42C	109.5
H13A—C13—H13C	109.5	H42A—C42—H42B	109.5
H13B—C13—H13C	109.5	H42A—C42—H42C	109.5
Si1S—C13S—H13D	109.5	H42B—C42—H42C	109.5
Si1S—C13S—H13E	109.5	Si3—C43—H43A	109.5
Si1S—C13S—H13F	109.5	Si3—C43—H43B	109.5
H13D—C13S—H13E	109.5	Si3—C43—H43C	109.5
H13D—C13S—H13F	109.5	H43A—C43—H43B	109.5
H13E—C13S—H13F	109.5	H43A—C43—H43C	109.5
S3—C14—S4	105.04 (16)	H43B—C43—H43C	109.5
C15—C14—S3	107.6 (2)	S12—C44—S11	105.83 (16)
C15—C14—S4	109.3 (2)	C45—C44—S11	110.4 (2)
C16—C14—S3	111.4 (2)	C45—C44—S12	111.5 (2)
C16—C14—S4	110.9 (2)	C45—C44—C46	111.7 (3)
C16—C14—C15	112.2 (3)	C46—C44—S11	109.1 (2)
C14—C15—H15A	109.5	C46—C44—S12	108.1 (2)
C14—C15—H15B	109.5	C44—C45—H45A	109.5
C14—C15—H15C	109.5	C44—C45—H45B	109.5
H15A—C15—H15B	109.5	C44—C45—H45C	109.5
H15A—C15—H15C	109.5	H45A—C45—H45B	109.5
H15B—C15—H15C	109.5	H45A—C45—H45C	109.5
C14—C16—H16A	109.5	H45B—C45—H45C	109.5
C14—C16—H16B	109.5	C44—C46—H46A	109.5
C14—C16—H16C	109.5	C44—C46—H46B	109.5
H16A—C16—H16B	109.5	C44—C46—H46C	109.5
H16A—C16—H16C	109.5	H46A—C46—H46B	109.5
H16B—C16—H16C	109.5	H46A—C46—H46C	109.5
C18—C17—C1	124.2 (3)	H46B—C46—H46C	109.5
C22—C17—C1	119.0 (3)	C8—S2—C4	97.9 (2)
C22—C17—C18	116.6 (3)		

### Hydrogen-bond geometry (Å, º)

**Table:**

*D*—H···*A*	*D*—H	H···*A*	*D*···*A*	*D*—H···*A*
O1—H1···S8	0.84	2.32	3.031 (2)	142
C9—H9*C*···S5	0.98	3.05	3.926 (5)	150
C12—H12*A*···S3	0.98	2.51	3.184 (16)	126
C13—H13*B*···S6^i^	0.98	2.85	3.734 (10)	150
C15—H15*B*···S11^ii^	0.98	3.00	3.866 (4)	148
C16—H16*C*···S5	0.98	3.00	3.912 (4)	155
C26—H26*C*···S6	0.98	2.68	3.364 (5)	127
C31—H31*A*···S12^ii^	0.98	2.81	3.435 (3)	123
C41—H41*A*···S10	0.98	2.87	3.508 (5)	123
C42—H42*C*···S10	0.98	2.60	3.291 (3)	128
C45—H45*C*···S1	0.98	2.96	3.867 (4)	155

Symmetry codes: (i) −*x*+2, −*y*+2, −*z*+2; (ii) −*x*+2, −*y*+2, −*z*+1.
